# Adjustable Phase-Amplitude-Phase Acoustic Metasurface for the Implementation of Arbitrary Impedance Matrices

**DOI:** 10.34133/research.0502

**Published:** 2024-10-08

**Authors:** Yu-Ze Tian, Zhuo-Run Wei, Yan-Feng Wang, Vincent Laude, Yue-Sheng Wang

**Affiliations:** ^1^ School of Mechanical Engineering, Tianjin University, 300350 Tianjin, China.; ^2^ School of Science, Tianjin University, 300350 Tianjin, China.; ^3^ National Key Laboratory of Vehicle Power System, 300350 Tianjin, China.; ^4^ Université de Franche-Comté, CNRS, Institut FEMTO-ST, F-25000 Besançon, France.; ^5^ Institute of Engineering Mechanics, Beijing Jiaotong University, Beijing 100044, China.

## Abstract

Impedance metasurfaces enable accurate regulation of acoustic fields. However, they can hardly supply a flexible response as such perfect operation is accompanied by stringent requirements on the design of unit cells. Actually, an arbitrary lossless and passive target impedance matrix requires the tuning of 3 independent real parameters. The set composed of a reflection phase, a transmission amplitude, and a transmission phase, enables the representation of an arbitrary impedance matrix, possibly possessing singular elements. In this paper, a mechanism of phase-amplitude-phase modulation (PAP modulation) is developed for the generic design of the unit cells of acoustic impedance metasurfaces. Adjustable acoustic impedance metasurfaces are further available under this framework. An impedance unit with 3 mobile parts is designed based on this idea. The assembled metasurface can handle different incidences for acoustic field manipulation at a given frequency. Beam steering and beam splitting are considered as demonstration examples and are verified by numerical simulation and experiment. PAP modulation enriches the design of acoustic impedance metasurfaces and extends the range of application of impedance theory.

## Introduction

Acoustic metasurfaces, a subset of acoustic metamaterials [1–5], have aroused great interest these days. They greatly facilitate wave field manipulation in airborne acoustics [6], transmedia acoustics [7], underwater acoustics [8], and even fluid dynamics [9] through compact structural design. Several theoretical approaches have been proposed to sustain this concept [10–13]. The recently proposed impedance theory, which is now attracting wide attention, imposes strict power flow conservation, in contrast to former approaches [14–16]. Various functions have been implemented rigorously with acoustic impedance metasurfaces, for instance beam steering [15], beam splitting [17,18], orbital angular momentum generation [19], plane-cylinder wave conversion [20–22], and encrypted information storage [23].

Impedance theory was first proposed in electromagnetism, with the benefit that parasitic scattering can be eliminated provided the boundary impedance is rationally tailored [14]. Transplanted to acoustics by Díaz-Rubio and Tretyakov. [15], impedance models for both reflective and refractive metasurfaces were established. A reflective impedance metasurface with simple slot units was in particular designed. Nonlocal power flow, however, seemed hardly avoidable due to the occurence of real values in the boundary impedance. In order to reduce complexity, curved metasurfaces were then investigated under a power flow conformal strategy, using similar slot units [24]. Bending the metasurface in the light of the distribution of the power flow enabled the boundary impedance to remain purely imaginary. The resulting metasurface could then be designed independently with a discretized structure of elementary unit cells. Li et al. [25] proposed a structure with Helmholtz resonators for the design of refractive metasurfaces. With this solution, the impedance matrix of the interface can be approached by independent unit cells composed of 4 cascaded resonators with inhomogeneous sound capacities. This approach was later generalized by Tian et al. [18] within the framework of integral equivalence. Actually, it can be proven that any pair of acoustic fields can be passively connected as long as global power flow conservation is imposed.

Nonetheless, there is so far no clear solution to design a structure implementing an arbitrary impedance matrix, especially in the case of transmission metasurfaces. Helmholtz resonator units may cover a certain range of impedance matrices [25], but it remains difficult to infer without resorting to optimization what geometric parameters are sufficient for achieving a target impedance matrix. In addition, although impedance theory is, in principle, as functional as the generalized Snell’s law is, it somehow lacks its flexibility. Once the impedance metasurface is fabricated, indeed, the incident and the reflected or refracted wave fields are fully determined [23]. Perfect power flow manipulation hence can only be observed under preset operating conditions. The requirement on a flexible response cannot be fulfilled by the current Helmholtz resonators, as well. Adjustable structures like those based on the generalized Snell’s law [26–32] are desirable so that the impedance matrix can be adjusted according to the target function, which has not been achieved yet.

In this paper, a mechanism of phase-amplitude-phase modulation (PAP modulation) is developed for the design of unit cells of acoustic impedance metasurfaces. It is first argued that the number of independent design parameters is 3. It is proved that any conservative impedance matrix can be approached by modulating the transmitted and reflected waves from one side. Based on this mechanism, an adjustable unit with 2 phase modulators and an amplitude modulator is designed. The assembled metasurface provides target wave manipulation under different incidence angles at a given frequency. Beam steering with 2 different operation angles and beam splitting are demonstrated by numerical simulation based on impedance theory. Scattering is almost undetectable in all 3 considered scenarios. Two groups of samples are fabricated and show nearly perfect experimental operation.

## Results

### PAP modulation and physical realization

**Fig. 1.**
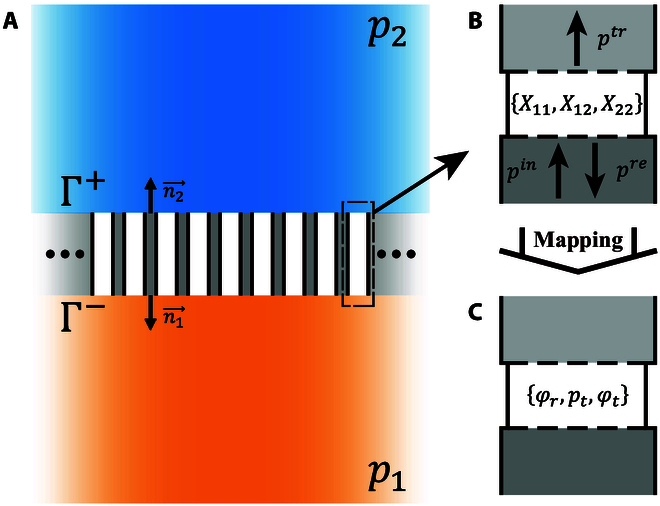
Schematic representation of impedance theory. (A) An impedance metasurface connecting 2 acoustic fields *p*_1_ and *p*_2_ is discretized into unit cells for design purposes. Adjacent unit cells are rigidly isolated to avoid crosstalk. (B) The impedance components {*X*_11_, *X*_12_, *X*_22_} of each unit cell can be monitored independently under plane wave incidence. (C) After establishing the mapping between the impedance matrix *Z* = ı*X* and the transmission and reflection coefficients, impedance unit cells are characterized by the real triplet {*φ_r_*, *p_t_*, *φ_t_*}.

We start by recalling certain essential results on impedance unit cells of acoustic metasurfaces. Generally, a transmission metasurface is regarded as the connection between 2 acoustic fields *p*_1_ and *p*_2_, each obeying a scalar Helmholtz equation [18], as shown in Fig. 1A. A passive and lossless impedance matrix is expressed as

$$\left[\begin{array}{c} p_{1}\\ p_{2} \end{array}\right]=ı\left[\begin{array}{cc} X_{11} & X_{12}\\ X_{21} & X_{22} \end{array}\right]\left[\begin{array}{c} -n_{1}\cdot v_{1}\\ -n_{2}\cdot v_{2} \end{array}\right]\mathrm{on}\;\Gamma ,$$ (1)

where ı is imaginary unit, **v***_i_* is the local velocity, and **n***_i_* is the normal vector to the interface entering the region (*i* = 1, 2). Diagonal components must be equal, *X*_12_ = *X*_21_, to ensure the continuity of normal power flow [23]. Unit cells arise from the discretization of the metasurface and each supply a specific impedance matrix determined by its spatial position and Eq. 1. Adjacent units should be rigidly isolated to ensure the absence of crosstalk and hence independent design. The tangential dimension must be deeply subwavelength, so that there is only one mode of propagation inside the unit cell at a given frequency. As a consequence, the impedance matrix *Z* = ı*X* is defined uniquely under any excitation and can be detected by coupling into a waveguide [17].

The unit cell is described by a set of 3 independent variables, e.g., {*X*_11_, *X*_12_, *X*_22_}. Assigning an obvious physical meaning to these impedance elements is, however, not immediate. Instead, from the set of incident, transmitted, and reflected waves (*p^in^*, *p^tr^*, and *p^re^* in Fig. 1B) [18], the impedance matrix can be written

$$\begin{array}{l} X=\left[\begin{array}{cc} X_{11} & X_{12}\\ X_{21} & X_{22} \end{array}\right]\\ \;=\frac{Z_{0}}{D}\left[\begin{array}{cc} \text{cos}\varphi_{t}+\frac{p_{r}}{p_{i}}\text{cos}\left(\varphi_{r}-\varphi_{t}\right) & \frac{p_{i}^{2}-p_{r}^{2}}{p_{t}p_{i}}\\ \frac{p_{t}}{p_{i}} & \text{cos}\varphi_{t}-\frac{p_{r}}{p_{i}}\text{cos}\left(\varphi_{r}-\varphi_{t}\right) \end{array}\right] \end{array}$$ (2)

$$D=\frac{p_{r}}{p_{i}}\text{sin}\left(\varphi_{r}-\varphi_{t}\right)+\text{sin}\varphi_{t},$$ (3)

where *p_i_*, *p_t_*, and *p_r_* are the amplitudes of incident, transmitted, and reflected waves, respectively, *φ_r_* and *φ_t_* are the reflection and transmission phases referred to the incident wave, and *Z*_0_ is the characteristic impedance of the background medium. *X*_12_ and *X*_21_ are equal under the condition of power flow conservation, $p_{i}^{2}=p_{r}^{2}+p_{t}^{2}$. The set of 3 independent real quantities {*φ_r_*, *p_t_*, *φ_t_*} can be taken as representative of the impedance matrix. Furthermore, an inverse mapping is

$$\begin{array}{l} \frac{p_{r}}{p_{i}}e^{ı\varphi_{r}}=\frac{\left(ıX_{11}-Z_{0}\right)\left(ıX_{22}+Z_{0}\right)+X_{12}X_{21}}{\left(ıX_{11}+Z_{0}\right)\left(ıX_{22}+Z_{0}\right)+X_{12}X_{21}},\\ \frac{p_{t}}{p_{i}}e^{ı\varphi_{t}}=\frac{2ıX_{12}Z_{0}}{\left(ıX_{11}+Z_{0}\right)\left(ıX_{22}+Z_{0}\right)+X_{12}X_{21}}. \end{array}$$ (4)

Accordingly, there is always a unique couple of complex transmission and reflection coefficients for any impedance matrix.

The mapping above provides us with an opportunity to characterize the impedance matrix using {*φ_r_*, *p_t_*, *φ_t_*}, as shown in Fig. 1C. An arbitrary impedance matrix can be therefore be achieved uniquely by simply modulating the amplitudes and phases of the transmitted and reflected waves, excited from one side of the metasurface. As a consequence, unit cell design can be decoupled into 3 structures that supply independent modulation on the reflection phase, the reflection/transmission amplitude, and the transmission phase. We name such a mechanism PAP modulation of the impedance matrix.

Interestingly, even singular impedance matrices fall under this framework. For instance, the impedance matrix is singular if *φ_t_* = *mπ*(*m* ∈ ℤ) with no reflection (*p_r_* = 0). At this point, the impedance unit actually provides a phase difference of *π* or nothing but continuity, which can be easily achieved by a phase modulator. Another special case is *φ_t_* = *mπ*(*m* ∈ ℤ) with *φ_r_* − *φ_t_* = *nπ*(*n* ∈ ℤ). The impedance matrix is then singular as well. This situation can perfectly occur with PAP modulation.

**Fig. 2.**
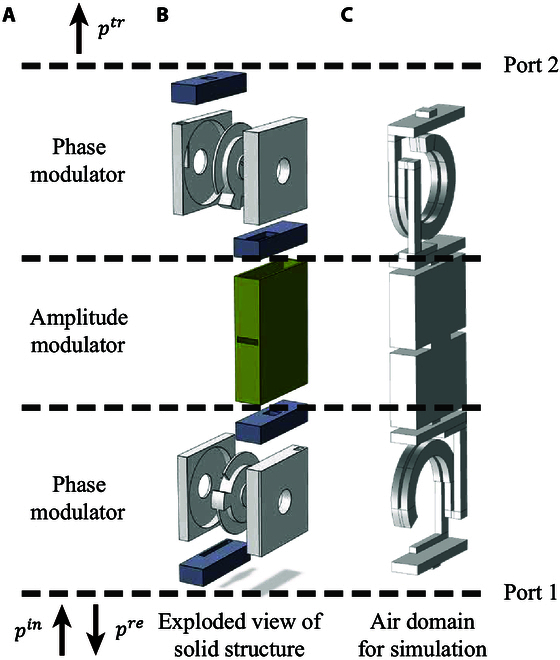
Schematic representation of the designed adjustable acoustic impedance unit. (A) PAP modulation. (B) Exploded view of the solid structure consisting of 2 phase modulators (white parts) with 4 resonators (gray parts) and one amplitude modulator (yellow part). (C) Air domain considered in numerical simulations.

In contrast to the previous design of 4 series-connected Helmholtz resonators [25], only 3 sections are required for PAP modulation, as shown in Fig. 2A. One can easily design a structure with a specific impedance matrix by connecting 2 phase modulators through an amplitude modulator. Ideally, the transmission and reflection amplitudes would be uniquely controlled by the amplitude modulator when designing the 2 phase modulators to have a perfect transmittance [33]. The reflection phase is controlled by the lower phase modulator, and the transmission phase is jointly controlled by both phase modulators. The selection of the structural geometry is made easier, as there exist abundant structures supplying a phase shift with a complete transmission [34,35], while the amplitude modulator can be regarded as a simple insertion. Theoretically, impedance unit cells can be made ultrathin as well if they are based on the idea of space coiling [36,37], but the latter choice is not compulsory.

Furthermore, adjustable acoustic impedance metasurfaces are available under the mechanism of PAP modulation. A unit cell with 3 adjustable parts is designed as an example. The operating frequency is selected as *f*_0_ = 3, 500 Hz, or wavelength in air *λ*_0_ = *c*_0_/*f*_0_ = 9.8 cm. The tangential dimension of the unit cells is *d_u_* = 1.12 cm, or about *λ*_0_/9. The exploded view is depicted in Fig. 2B (see Supplementary Materials Section S1 for details of the optimized geometry and transmission characteristics of the designed unit). The 2 phase modulators (white parts) are images in a central symmetry and are connected through an amplitude modulator (yellow part). Two resonators (gray parts) are connected in series at both ends of each phase modulator. High transmittance for phase difference modulation is guaranteed by optimization of the 2 resonators. The propagation distance (the air domain in Fig. 2C) changes accordingly when rotating the middle knob. The modulators then support linear and continuously adjustable phase differences. The amplitude modulator, located between the 2 phase modulators, is a cuboid cavity with a central opening for insertion (not drawn for simplicity). The amplitude of transmittance can be modulated by changing the cavity depth. The reflection phase for waves incident from below is uniquely controlled by the lower modulator, while the transmission phase is jointly controlled by both modulators. Consequently, the impedance matrix of the unit can be adjusted by coordinating these 3 components. The array direction of unit cells is perpendicular to the rotation plane of the knob. The upper and lower ports are connected to the acoustic fields directly.

### Simulation and characterization

**Fig. 3.**
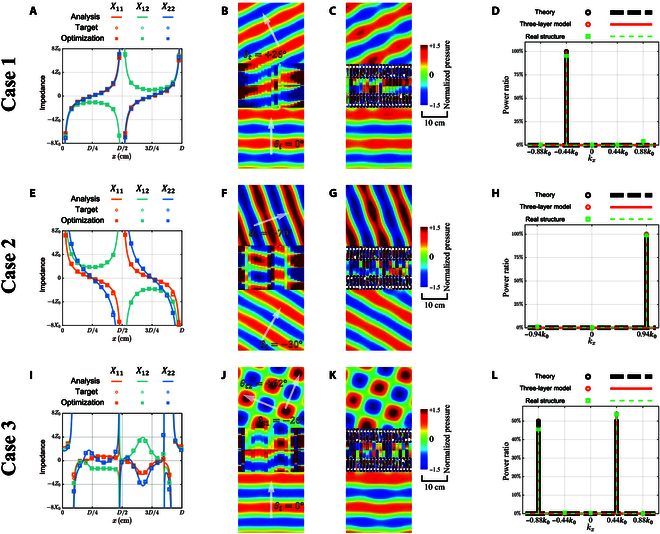
Numercial simulation of adjustable impedance metasurfaces. Case 1: (A) Interface impedance, (B) simulation by 2D 3-layer model, and (C) simulation by 3D FEA for beam steering with *θ_i_* = 0° and *θ_t_* =  + 26°, (D) quantitative evaluation provided by 2D FFT. Case 2: (E) Interface impedance, (F) simulation by 2D 3-layer model, and (G) simulation by 3D FEA for beam splitting with *θ_i_* =  − 30° and *θ_t_* =  − 70°, (H) quantitative evaluation provided by 2D FFT. Case 3: (I) Interface impedance, (J) simulation by 2D 3-layer model, and (K) simulation by 3D FEA for beam splitting with *θ_i_* = 0°, *θ*_*t*1_ =  + 62°, and *θ*_*t*2_ =  − 26°_,_ (L) quantitative evaluation provided by 2D FFT.

Three cases are examined next to check the operation of the designed unit cells when assembling a metasurface. Twenty unit cells are arrayed together with repetition distance *D* = 20*d_u_*. The case of beam steering under normal incidence (*θ_i_* = 0°) is demonstrated first. The refraction angle is set to *θ_t_* =  + 26°. The continuously varying impedance elements are plotted as solid lines in Fig. 3A (see Supplementary Materials Section S2 for a detailed calculation of interface impedance). Twenty sampling points (circles in Fig. 3A) are uniformly selected with an interval of *d_c_* for numerical simulation by the 3-layer numerical model [15] (see Simulations). Results are shown in Fig. 3B. It can be observed that almost all power is redirected toward the target direction. Limited spurious scattering occurs due to discretization of unit cells.

Unit cells are then adjusted to approach the required impedance matrix with the help of optimization. The rotation angles of the knobs in the 2 phase modulator and the depth of the insertion in the amplitude modulator can then be obtained (see Supplementary Materials Section S3 for detailed parameters). The impedance provided by the designed structure is plotted with square points in Fig. 3A. Assembling the unit cells into a metasurface, finite element analysis (FEA) of wave transmission is presented in Fig. 3C (see Simulations). Little degradation is observed compared with the 3-layer numerical model. Two-dimensional Fourier transform (2D FFT) of the transmitted field is adopted for a quantitative evaluation and is presented in Figs. 3D. It can be noticed that the efficiency obtained using the 3-layer numerical model (orange solid line) is close to 100% and basically consistent with the theoretical value (black dashed line). The numerical result obtained considering practical structure (green dashed line) is consistent with the result for the 3-layer model as well. Spurious power is allocated to other directions, which we attribute to the discretization of unit cells.

Beam steering with large refraction angles is, in principle, an advantage of metasurfaces based on impedance theory compared to those based on the generalized Snell’s law [15]. A transmission angle *θ_t_* =  − 70° under an incidence angle of *θ_i_* = − 30° is considered as an example. The interface impedance provided by theoretical analysis is shown in Fig. 3E, together with the result of the optimization of the unit cells. The adjustable impedance metasurface approaches the target impedance successfully. Numerical simulation of the acoustic fields by the 3-layer model and FEA are shown in Fig. 3F and G. Beam steering at a large angle is obtained with limited spurious scattering. A quantitative evaluation is obtained by 2D FFT in Fig. 3H. The 3 lines are almost overlapping, and the efficiency is exactly 100%.

The case of beam splitting is considered next to illustrate the versatility of the design. It is known the impedance theory supports perfectly splitted beams with different angles [17], in contrast to the generalized Snell’s law [38,39]. A set of opposing surface waves should be excited on the incident side to balance the power flow. Thus, the impedance metasurface simultaneously manipulates the fields on both the incident and transmitted sides, which goes beyond the generalized Snell’s law. Two splitted beams are targeted under normal incidence with the same amplitudes and transmission angles *θ*_*t*1_ =  + 62° and *θ*_*t*2_ =  − 26°. The required impedance distribution is more complex than for a single output beam, as shown in Fig. 3I. The interface impedances provided by the adjustable units, anyway, still coincide with the target values. Satisfying operation is obtained in numerical simulations by the 3-layer numerical model (Fig. 3G) and FEA (Fig. 3K). Almost all of the power flow is transmitted and allocated correctly as shown in Fig. 3L. Only a slight unevenness of the target directions occurs due to the discretization of unit cells.

We emphasize that the complete set of impedance matrices, and not the local transmission and reflection coefficients, is required to evaluate the performance of the adjustable unit cell, although the latter is approached locally through PAP modulation. This is because both reflected and transmitted fields are actually controlled by the interface impedance provided by the whole metasurface (see Eq. 1). The local transmittance of a particular unit cell does not have a substantial impact on the entire wave field in the framework of impedance theory. As an example, the interface impedance of the metasurface designed for anomalous refraction corresponds to a scattering matrix with component ∣*S*_12_ ∣  ≤ 1. This indicates that the unit cells that compose this metasurface always show a local transmittance that is less than 1 (see Supplementary Materials Section S2 for a detailed proof). However, they collectively provide the required impedance relationship of Eq. 1 at the boundary, stably connecting the preset incident and transmitted fields. As a result, manipulation of the transmitted field remains perfect as shown in Fig. 3.

Moreover, the uniform width of the unit cells of the metasurfaces exhibited in this paper is only chosen for convenience. The assembled metasurface always provides perfect wave manipulation even if the width of unit cells is not uniform. The adaptability of impedance theory is therefore greatly promoted as different goals can be achieved under arbitrary incidence.

### Experimental realization and measurements

**Fig. 4.**
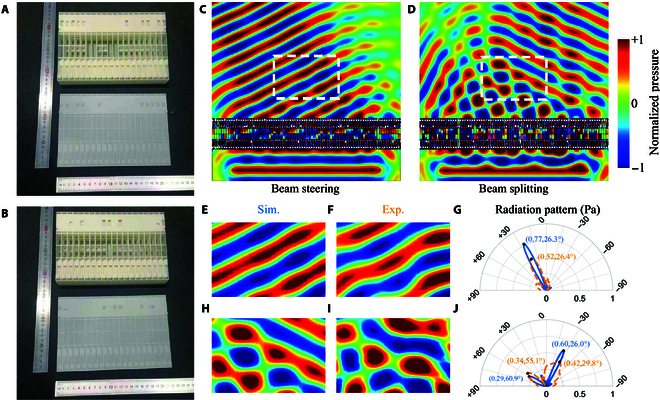
Experimental verification of adjustable impedance metasurface. A period of the metasurface sample is shown for (A) Case 1 and (B) Case 3. Simulation results for (C) Case 1 and (D) Case 3 are given for the metasurface excited by a finite-width beam. The acoustic field of Case 1 in the measured area is given for (E) numerical simulation and (F) experiment and (G) corresponding radiation pattern. The acoustic field of Case 3 in the measured area is given for (H) numerical simulation and (I) experiment and (J) corresponding radiation pattern.

Experiments were conducted to validate the operation of the designed metasurfaces (see Experiments). Metasurface samples were fabricated by 3D printing, one period at a time for simplicity. Four periods are included in each metasurface during experiments. Photographs of the samples corresponding to Case 1 (beam steering) and Case 3 (beam splitting) are shown in Fig. 4A and B. Each sample is composed of a main body with a thickness of 3.3 cm and a cover with a thickness of 0.2 cm introduced for convenience of manufacturing. Numerical simulations by FEA following experimental settings are presented in Fig. 4C and D for comparison purposes (see Simulations). Close to perfect operation is observed visually in both cases. Some distortions occur at the edges of the refractive beam in Fig. 4C due to the limited width of the metasurface. The splitted beams in Fig. 4D share the same amplitude, as expected, although different transmission angles result in different beam widths.

Figure 4 further provides close-up views at the acoustic fields. The region corresponding to the measurement area available during experiments is overlaid with a white dashed box in Fig. 4C and D. The simulated pressure field of Case 1 is presented in Fig. 4E, whereas the experimental pressure field is presented in Fig. 4F. All color maps are normalized according to the average amplitude of the incident field. Good consistency is observed visually, except for some undesired disturbances in the experimental results. The radiation pattern is provided in Fig. 4G for quantitative evaluation (see Supplementary Materials Section S4 for a detailed definition). It can be seen from the main lobe in Fig. 4L that the metasurface for beam steering provides a refraction angle of *θ_t_* = 26^∘^ in both simulation and experiment. The amplitude of the main experimental lobe is reduced due to thermoviscous losses, but the beam direction is not affected dramatically. The situation is similar in the case of beam splitting shown in Figs. 4H and I. It should be noted that the amplitudes of the spitted beams are not the same in the far field as shown in Fig. 4J. This is owing to the difference in beam widths mentioned earlier. Their ratio is equal to the ratio of the cosine of splitting angle cos*θ*_*t*1_/ cos *θ*_*t*2_. In general, there are only slight deviations in amplitude and direction in the experimental result.

## Conclusion

In this paper, a mechanism termed PAP modulation has been developed for the design of the unit cells of adjustable acoustic impedance metasurfaces. It was proven that any impedance matrice can be approached by the modulation of transmission and reflection coefficients, with a total of 3 independent parameters. Each unit cell is designed to be composed of 3 mobile parts and can implement an arbitrary impedance matrix. Functions such as beam steering and beam splitting can be realized efficiently under arbitrary incidence. The operation was verified both by numerical simulation and by experiment. Theoretically, stepless adjustment of beam steering and beam splitting could be achieved as well through automation control, though the practical fabrication of adjustable structures might be a limitation as of today. More efficient and accurate manufacturing techniques will be the focus of future research. The work reported here is expected to enhance the versatility and applicability of acoustic impedance metasurfaces.

## Materials and Methods

### Simulations

The numerical simulations in Fig. 3B, E, and H are conducted with the 3-layer numeral model [15] in 2D, using the COMSOL Multiphysics Pressure Acoustics module. Each unit is composed of 2 waveguides with a length *l*_0_ = 7.45 cm and 3 interior impedance boundaries determined by

$$Z_{1}=ıX_{11}+ıX_{12}+ıZ_{0}\text{cot}\left(k_{0}l_{0}\right),$$ (5)

$$Z_{2}=2ıZ_{0}\text{cot}\left(k_{0}l_{0}\right)-\frac{Z_{0}^{2}}{ıX_{12}}{\text{sin}}^{-2}\left(k_{0}l_{0}\right),$$ (6)

$$Z_{3}=ıX_{22}+ıX_{21}+ıZ_{0}\text{cot}\left(k_{0}l_{0}\right).$$ (7)

Adjacent unit cells are isolated by an interior sound hard boundary. The incident and transmitted fields below and above the metasurface have a width equal to the metasurface period and a height 2.1*λ*_0_. Periodic boundary conditions with Bloch–Floquet periodicity are applied on the left and right boundaries of the model. The Bloch *k*-vector is set as *k* = (*k*_0_ sin *θ_i_*, −*k*_0_ cos *θ_i_*). Two perfectly matched layers with a thickness 0.6*λ*_0_ are connected to the upper and lower boundaries of the model to avoid numerical reflections. The mesh is set with a maximum size of *λ*_0_/10 and a minimum size of *λ*_0_/20 for convergence. An additional refinement of the mesh is performed near the impedance boundaries. A plane wave with unit amplitude *p_i_* = 1 Pa and incidence angle *θ_i_* is set as the background pressure field.

The numerical simulations in Fig. 3C, F, and I are conducted with practical structures in 3D. The incident and transmitted fields below and above the metasurface have a width equal to the metasurface period and a height 2.1*λ*_0_. The thickness of the model is consistent with the thickness of the metasurface (3.5 cm). Periodic boundary conditions with Bloch–Floquet periodicity are applied on the left and right sides of the model. The Bloch *k*-vector is set as *k* = (*k*_0_ sin *θ_i_*, −*k*_0_ cos *θ_i_*, 0). Two perfectly matched layers with a thickness 0.6*λ*_0_ are connected to the upper and lower boundaries of the model to avoid numerical reflections. All other boundaries are set to sound hard boundaries. The mesh is generated with a maximum size of *λ*_0_/10 and a minimum size of *λ*_0_/20 for convergence. An additional refinement of the mesh is performed near the metasurface. A plane wave with unit amplitude *p_i_* = 1 Pa is set as the background pressure field.

The simulations in Fig. 4C and D are set according to the experimental environment. Four periods of the metasurface are included. A line source with a length of 70 cm, unit amplitude, and an operating frequency of 3,500 Hz is placed in the incident field at a distance of 10 cm from the metasurface. The transmitted and incident regions above and below the metasurface are set with a length of 89.6 cm, a height of 3.5 cm, and a width of 55 and 15 cm, respectively. Both regions are surrounded by perfectly matched layers with a width of 10 cm to avoid reflections.

### Experiments

The real experimental setup is shown in Supplementary Materials Fig. S6. The solid part of the designed metasurfaces is composed of a photosensitive resin which can be regarded as rigid in air. Four periods of the metasurface are included and placed in a 2D single-mode waveguide with a thickness of 3.5 cm. A line source with a length of 70 cm and an operating frequency of 3,500 Hz is placed at a distance of 10 cm from the metasurface. The whole experimental environment is surrounded by foam wedges to avoid reflections. A rectangular area with a length of 30 cm and a width of 20 cm, placed 10 cm away from the metasurface, is selected as the measuring area. The signals generated and collected in the experiment are controlled by a B&K 3160-A-042 control module connected to a computer. There are 30 × 20 evenly distributed points with a sampling spacing of 1 cm.

## Data Availability

All data needed to evaluate the conclusions in the paper are present in the paper and/or the Supplementary Materials. Additional data related to this paper may be requested from the authors.

## Supplementary Materials

Supplementary 1 Sections S1 to S4 Figs. S1 to S6 Tables S1 to S3
