# Supplementary material for: Adjustable Phase-Amplitude-Phase Acoustic Metasurface for the Implementation of Arbitrary Impedance Matrices
Source: Research (Wash D C). 2024 Oct 8;7:0502. doi: 10.34133/research.0502 (PMC11458265; doi:10.34133/research.0502)
Supplement: Supplementary 1 — Sections S1 to S4 Figs. S1 to S6 Tables S1 to S3 [file research.0502.f1.pdf]

Supplemental Information for Adjustable phase-amplitude-phase acoustic metasurface for the implementation of arbitrary impedance matrices

Yu-Ze Tian,[1] Zhuo-Run Wei,[2] Yan-Feng Wang,[1,3,*] Vincent Laude,[4] and Yue-Sheng Wang[1,5]

[1]*School of Mechanical Engineering, Tianjin University, 300350 Tianjin, China*
[2]*School of Science, Tianjin University, 300350 Tianjin, China*
[3]*National Key Laboratory of Vehicle Power System, 300350 Tianjin, China*
[4]*Université de Franche-Comté, CNRS, Institut FEMTO-ST, F-25000 Besançon, France*
[5]*Institute of Engineering Mechanics, Beijing Jiaotong University, Beijing 100044, China*

SECTION S1. GEOMETRY OF THE DESIGNED ADJUSTABLE ACOUSTIC IMPEDANCE UNIT

There are three variable parameters in each adjustable impedance unit cell, as shown in Fig. 1(a), including $\theta_{\rm up}$ in the upper phase modulator [Fig. 1(b)], $d_{\rm in}$ in the amplitude modulator [Fig. 1(c)], and $\theta_{\rm down}$ in the lower phase modulator [Fig. 1(d)]. In te ideal case that the transmittance of two phase modulators approaches 100%, the transmission and reflection amplitudes are uniquely controlled by the amplitude modulator. Transmittance is 0% when the insertion cuts the amplitude modulator in half. Therefore, the amplitude modulator can definitely cover the amplitude modulation range from 0% to 100%. Hopefully, modulation of phase and amplitude can be decoupled. Indeed, the reflection phase for incidence from below is uniquely controlled by $\theta_{\rm down}$ and the transmission phase is then jointly controlled by $\theta_{\rm up}$ and $\theta_{\rm down}$. It can then be concluded from Eq. (2) of the main text that arbitrary impedance matrices are available if both phase modulators can provide a phase modulation covering the range from 0 to 2π.

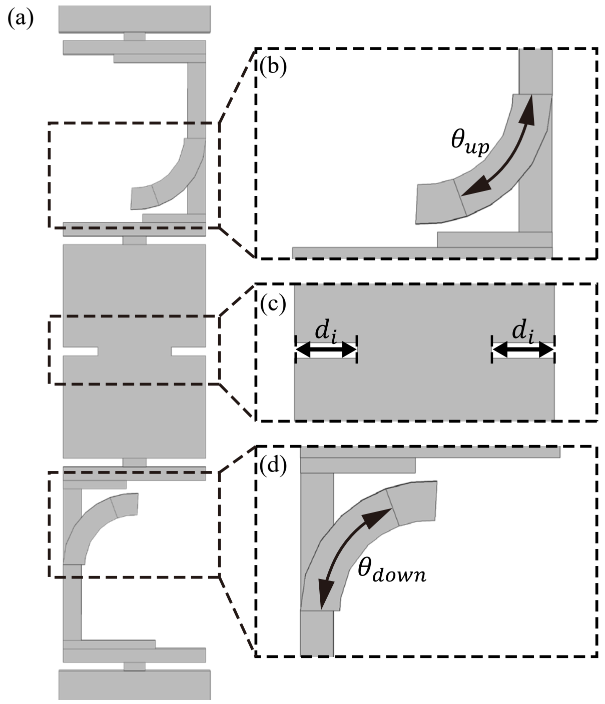

FIG. 1. The air domain of the adjustable acoustic impedance unit in simulation: (a) overall view, (b) the upper phase modulator, (c) the amplitude modulator, (d) the lower amplitude modulator.

Air with a mass density of $\rho_0 = 1.18$ kg/m^3 and a speed of $c_0 = 346$ m/s is adopted as the background medium under an operating frequency of $f_0 = 3500$Hz. The schematic diagram and the geometrical parameters of the phase modulator (white part) and the two resonators (gray parts) are shown in Fig. 2. Phase modulation is achieved by changing the propagation distance with the central knob. Two resonators are connected at the upper and lower sides to ensure a high transmittance. All parameters are optimized using a genetic algorithm taking minimum transmittance as the fitness value. The schematic diagram and the geometrical parameters of the amplitude modulator are shown in Fig. 3 as well. Two openings are located on both sides to adjust the insertion amplitude.

The power flow transmittance and the transmission phase of the optimized phase modulator with two resonators are plotted in Fig. 4(a). Phase modulation is basically linear and covers $0-2\pi$ with a minimum power flow transmittance of 84%. An arbitrary phase difference from 0 to 2π can thus be provided. Transmittance can be further increased after the two phase modulators are connected through the amplitude modulator. Two examples with $\theta_{up} = \theta_{down} = 0°$ and $\theta_{up} = \theta_{down} = 250°$ are shown in Fig. 4(b) and Fig. 4(c). The amplitude modulator actually becomes an additional resonator as the transmittance of both phase modulators is less than 100%. As the depth of the insertion increases, transmission first increases to 100% and then decreases to 0%, which leads to a full coverage of the amplitude range. In addition, phase modulation does not change significantly in this process except for a phase jump at the isolated point with 100% transmittance and no reflection. Consequently, coupling between phase modulation and amplitude modulation is relatively weak.

* wangyanfeng@tju.edu.cn

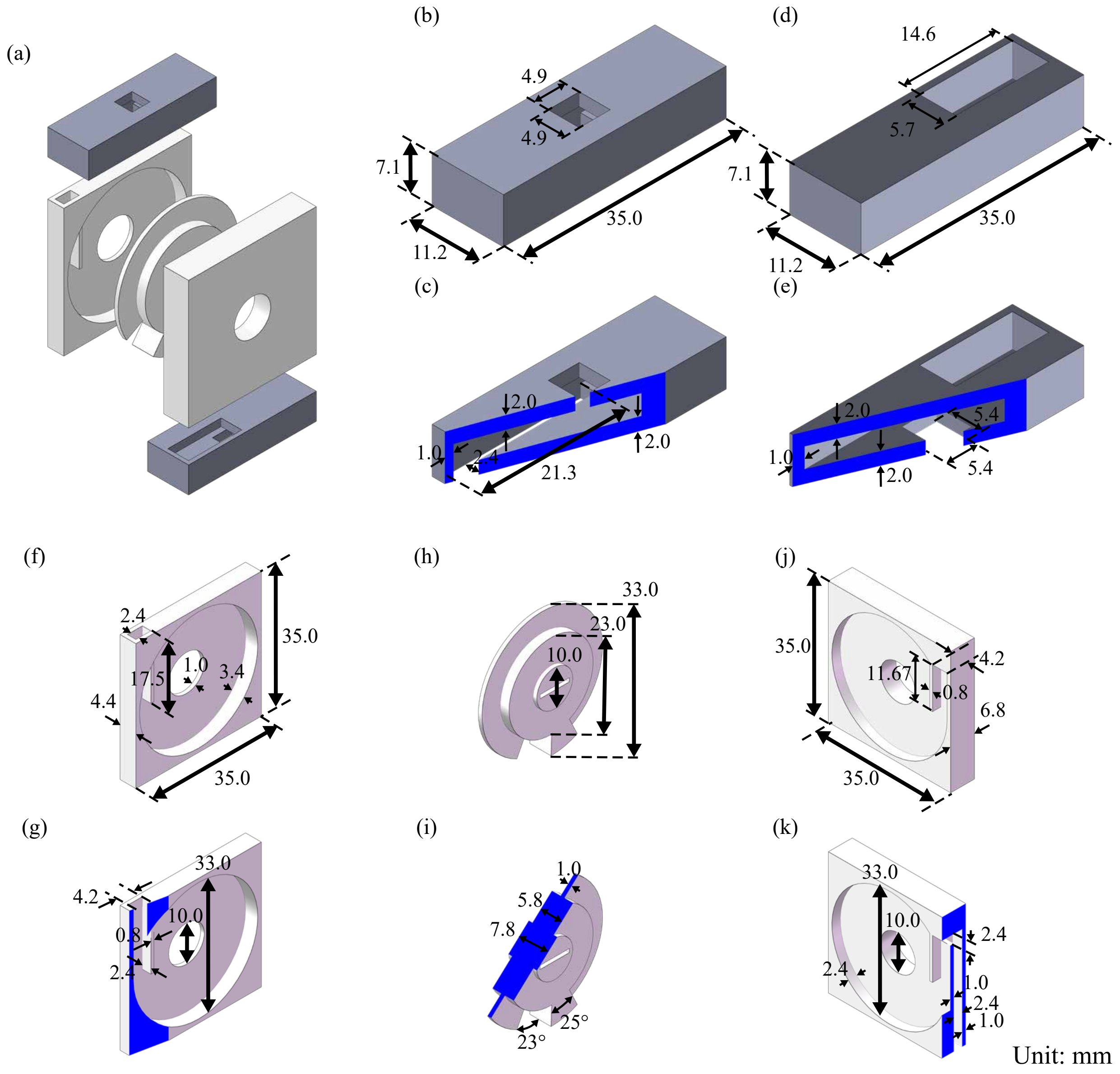

FIG. 2. Schematic diagram and geometric parameters of the phase modulator (white part) with two resonators (gray part): (a) explosion view of the whole solid structure, (b) overall and (c) sectional view of the upper resonator, (d) overall and (e) sectional view of the lower resonator, (f) overall and (g) sectional view of the left part of the phase modulator, (h) overall and (i) sectional view of the knob in the phase modulator, (j) overall and (k) sectional view of the right part of the phase modulator.

SECTION S2. SIMULATION AND THREE-LAYER NUMERAL MODEL

In order to define an impedance metasurface that includes a beam steering function, the incident and transmitted pressure fields are set as

$$p_1 = p_i e^{-\imath(k_0 \sin\theta_i x + k_0 \cos\theta_i y)}, \tag{1}$$

$$p_2 = p_t e^{-\imath(k_0 \sin\theta_t x + k_0 \cos\theta_t y)}, \tag{2}$$

with the velocity fields

$$\mathbf{v}_1 = -\frac{p_i}{Z_0} e^{-\imath(k_0 \sin\theta_i x + k_0 \cos\theta_i y)} \times \left(-\sin\theta_i \mathbf{e}_x - \cos\theta_i \mathbf{e}_y\right), \tag{3}$$

$$\mathbf{v}_2 = -\frac{p_t}{Z_0} e^{-\imath(k_0 \sin\theta_t x + k_0 \cos\theta_t y)} \times \left(-\sin\theta_t \mathbf{e}_x - \cos\theta_t \mathbf{e}_y\right). \tag{4}$$

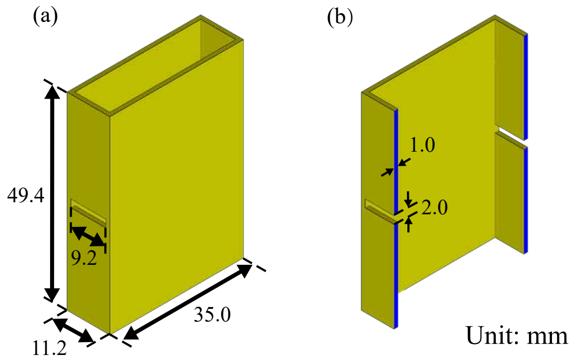

FIG. 3. Schematic diagram of the amplitude modulator (a) overall and (b) sectional view.

The power flow vector reads

$$\mathbf{I}_i = \frac{1}{2}\mathrm{Re}\left[p_i \mathbf{v}_i^*\right], \qquad i = 1, 2, \tag{5}$$

For convenience, $\Gamma : y = 0$ is adopted as the impedance interface. For a passive connection that strictly provides continuous power flow with no internal source or loss[1, 2], the normal power flow on Γ obeys

$$\mathbf{n}_1 \cdot \mathbf{I}_1|_\Gamma + \mathbf{n}_2 \cdot \mathbf{I}_2|_\Gamma = 0, \tag{6}$$

where $\mathbf{n}_i$ is the normal vector to the interface entering region $i = 1, 2$. Substituting Eqs. (1-5) into Eq. (6), a conservation condition can be obtained:

$$\frac{p_t}{p_i} = \sqrt{\frac{\cos\theta_i}{\cos\theta_t}}. \tag{7}$$

The impedance relationship on Γ is given by

$$\begin{bmatrix} p_1 \\ p_2 \end{bmatrix} = Z \begin{bmatrix} -\mathbf{n}_1 \cdot \mathbf{v}_1 \\ -\mathbf{n}_2 \cdot \mathbf{v}_2 \end{bmatrix} = \imath \begin{bmatrix} X_{11} & X_{12} \\ X_{21} & X_{22} \end{bmatrix} \begin{bmatrix} -\mathbf{n}_1 \cdot \mathbf{v}_1 \\ -\mathbf{n}_2 \cdot \mathbf{v}_2 \end{bmatrix} \text{ on } \Gamma. \tag{8}$$

The impedance matrix can then be solved as

$$Z = \imath \begin{bmatrix} \frac{Z_0}{\cos\theta_i}\cot\left[\Phi(x)\right] & \frac{Z_0}{\sqrt{\cos\theta_i\cos\theta_t}}\csc\left[\Phi(x)\right]) \\ \frac{Z_0}{\sqrt{\cos\theta_i\cos\theta_t}}\csc\left[\Phi(x)\right] & \frac{Z_0}{\cos\theta_t}\cot\left[\Phi(x)\right] \end{bmatrix}, \tag{9}$$

where

$$\Phi(x) = k_0 x\left(\sin\theta_i - \sin\theta_t\right). \tag{10}$$

The distributions of impedance components in Figs. 3 (a,d) in the main text are then obtained by substituting $\theta_i = 0°$ and $\theta_t = 26°$, and $\theta_i = 30°$ and $\theta_t = 70°$ into Eq. (9), respectively.

In order to define a impedance metasurface that includes a beam splitting function, the incident and transmitted pressure fields are set as

$$p_1 = p_i e^{-\imath k_0 y} + p_e e^{-\imath(k_e x + \alpha_e y)} + p_e e^{-\imath(-k_e x + \alpha_e y)}, \tag{11}$$

$$p_2 = p_{t1} e^{-\imath(k_0 x \sin\theta_{t1} + k_0 y \cos\theta_{t1})} + p_{t2} e^{-\imath(k_0 x \sin\theta_{t2} + k_0 y \cos\theta_{t2})}, \tag{12}$$

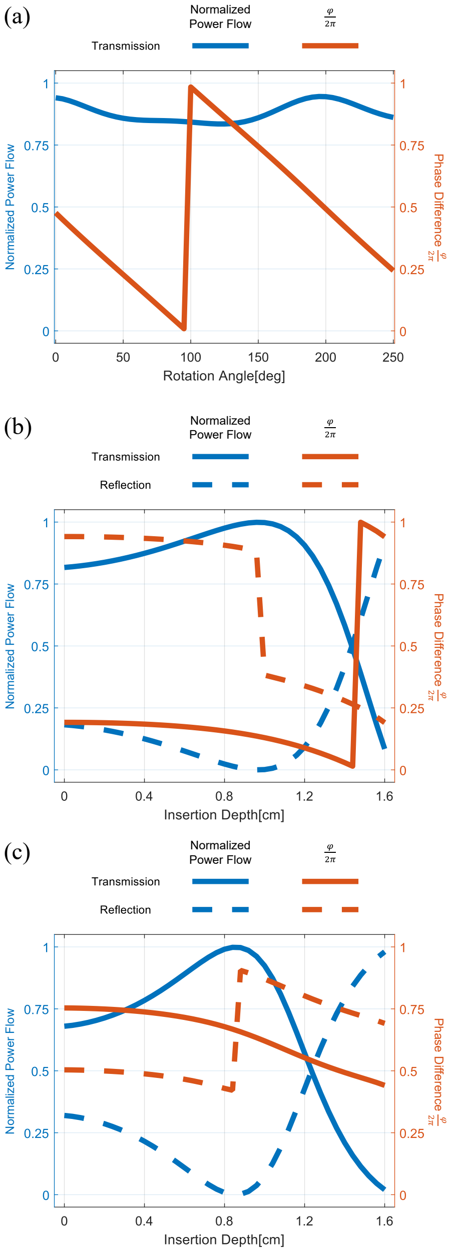

FIG. 4. Transmission characteristics of (a) the phase modulator with two resonators and the adjustable unit with (b) $\theta_{up} = \theta_{down} = 0°$ (c) $\theta_{up} = \theta_{down} = 250°$.

where θ_{t1} and θ_{t2} are the transmission angles. It should be noted that two evanescent waves with $k_e \in \mathbb{R}$ and $\alpha_e \in \mathbb{I}$ are introduced to balance the normal power flow[3, 4]. They should obey

$$k_e = k_0\left(\sin\theta_{t2} - \sin\theta_{t1}\right), \tag{13}$$

$$p_e = \frac{1}{2}p_{t1}p_{t2}\left(\cos\theta_{t1} + \cos\theta_{t2}\right). \tag{14}$$

Repeating the above process, the expressions of impedance components are given by

$$X_{11} = \left(p_{t1}\cos\left(\Phi_{t1}\right)\cos\theta_{t1} + p_{t2}\cos\left(\Phi_{t2}\right)\cos\theta_{t2}\right)\left(2p_e\cos\left(\Phi_e\right) + p_i\right) Z_0/D_0, \tag{15}$$

$$X_{12} = \left(2p_e\cos\left(\Phi_e\right) + p_i\right) Z_0/D_0, \tag{16}$$

$$X_{21} = \left[p_{t1}^2\cos\theta_{t1} + p_{t2}^2\cos\theta_{t2} + p_{t1}p_{t2}\left(\cos\theta_{t1} + \cos\theta_{t2}\right)\cos\left(\Phi_{t1} - \Phi_{t2}\right)\right] Z_0/D, \tag{17}$$

$$X_{22} = \left\{\left[p_{t1}\cos\left(\Phi_{t1}\right) + p_{t2}\cos\left(\Phi_{t2}\right)\right] - 2p_e\alpha_e/k_0\cos\left(\Phi_e\right)\left[p_{t1}\sin\left(\Phi_{t1}\right) + p_{t2}\sin\left(\Phi_{t2}\right)\right]\right\} Z_0/D_0, \tag{18}$$

$$D = \left[p_{t1}\sin\left(\Phi_{t1}\right)\cos\theta_{t1} + p_{t2}\sin\left(\Phi_{t2}\right)\cos\theta_{t2}\right] p_i + 2p_e\alpha_e/k_0\cos\left(\Phi_e\right)\left[p_{t1}\cos\left(\Phi_{t1}\right)\cos\theta_{t1} + p_{t2}\cos\left(\Phi_{t2}\right)\cos\theta_{t2}\right] \tag{19}$$

where

$$\Phi_{t1}\left(x\right) = k_0 x\sin\theta_{t1}, \tag{20}$$

$$\Phi_{t2}\left(x\right) = k_0 x\sin\theta_{t2}, \tag{21}$$

$$\Phi_e\left(x\right) = k_e x. \tag{22}$$

Considering $\theta_i = 0°$, $\theta_{t1} = +62°$ and $\theta_{t2} = -26°$, and imposing an amplitude ratio

$$\frac{p_{t1}}{p_i} = \frac{p_{t2}}{p_i} = \frac{1}{\sqrt{\left(\cos\theta_{t1} + \cos\theta_{t2}\right)/k_0}}, \tag{23}$$

the distribution of impedance components in Fig. 3 (g) in the main text are then obtained.

Although the overall performance of the impedance metasurface is ideally perfect, its unit cells actually have a local transmittance less than 1. We take the metasurface defined by Eq. (9) as an example to highlight this point. The forward transfer coefficient can be obtained from impedance components as [5]

$$S_{12}\left(x\right) = \frac{2Z_{12}Z_0}{\left(Z_{11} + Z_0\right)\left(Z_{22} + Z_0\right) - Z_{12}Z_{21}}. \tag{24}$$

Substituting Eq. (9) into Eq. (24), the amplitude can be calculated as

$$\begin{aligned} \left|S_{12}\right|\left(x\right) &= \frac{2}{\sqrt{\left(\sqrt{\cos\theta_i\cos\theta_t} + \frac{1}{\sqrt{\cos\theta_i\cos\theta_t}}\right)^2\sin^2\Phi\left(x\right) + \left(\sqrt{\frac{\cos\theta_i}{\cos\theta_t}} + \sqrt{\frac{\cos\theta_t}{\cos\theta_i}}\right)^2\cos^2\Phi\left(x\right)}} \\ &\leq \frac{2}{\sqrt{4\sin^2\Phi\left(x\right) + 4\cos^2\Phi\left(x\right)}} = 1. \end{aligned} \tag{25}$$

The maximum can be obtained if and only if $\cos\theta_i = \cos\theta_t = 1$, which corresponds to a transparent metasurface under normal incidence. Therefore, when a refractive impedance metasurface is designed in discrete unit cells, the local forward transfer coefficient at $x = x_0$ is $\left|S_{12}\left(x_0\right)\right| \leq 1$. If the output of the unit cell is taken out alone and checked by coupling into a waveguide [4], the transmittance is $T = \left|S_{12}\left(x_0\right)\right|^2 \leq 1$. However, manipulation of the transmission field remains ideal as shown in Figs. 3 in the main text. Therefore, it can be concluded that the performance of an impedance metasurface is actually controlled by the global interface impedance provided by the whole metasurface [see Eq. (1)] and not by the local transmission and reflection coefficients, although unit cells are designed based on PAP modulation.

SECTION S3. PARAMETERS OF PRACTICAL STRUCTURES

The parameters of the adjustable units in all three cases are obtained through parameter optimization with impedance matrix set as the objective function. Detailed parameters are presented in Tables I-III.

SECTION S4. EXPERIMENTAL ENVIRONMENT AND EXTERIOR FIELD CALCULATION

The experimental setup is shown in Fig. 5. The simulations in Fig. 6 are set according to the experimental environment. All values are normalized taking the incident field as a reference in order to present a quantitative evaluation. The field in the incidence region in Fig. 6(a) is simulated under the same setting but removing the metasurface. A close-up view is extracted from

TABLE I. Parameter configuration of the metasurface in Case 1.

Unit	θ_{up}[°]	d_i[cm]	θ_{down}[°]
1	206	1.03	188
2	196	0.84	208
3	11	1.00	198
4	214	1.04	4
5	218	1.06	213
6	17	1.06	220
7	22	1.07	223
8	228	1.02	234
9	33	0.91	242
10	124	0.01	177
11	66	0.10	254
12	44	0.91	252
13	58	0.56	60
14	93	0.00	244
15	76	0.00	65
16	178	1.01	168
17	180	0.94	177
18	195	1.01	168
19	179	0.88	196
20	190	0.36	194

TABLE II. Parameter configuration of the metasurface in Case 2.

Unit	θ_{up}[°]	d_i[cm]	θ_{down}[°]
1	156	0.73	32
2	170	0.39	216
3	140	1.08	19
4	173	0.00	195
5	134	1.14	209
6	133	1.12	201
7	132	1.10	194
8	216	0.91	100
9	226	1.18	250
10	126	1.07	180
11	124	1.10	174
12	128	1.06	153
13	127	1.00	146
14	213	1.32	223
15	212	1.33	11
16	209	1.34	208
17	209	1.34	199
18	4	1.36	194
19	175	0.59	22
20	177	0.26	16

TABLE III. Parameter configuration of the metasurface in Case 3.

Unit	θ_{up}[°]	d_i[cm]	θ_{down}[°]
1	221	0.90	178
2	156	0.80	225
3	78	1.51	47
4	20	1.50	49
5	36	0.78	78
6	131	0.43	207
7	120	0.83	219
8	131	0.62	209
9	234	0.81	82
10	52	1.64	153
11	202	0.00	245
12	220	1.22	223
13	131	0.98	146
14	131	0.99	145
15	17	1.09	236
16	179	1.33	252
17	133	1.44	44
18	168	0.59	12
19	15	1.04	175
20	6	0.39	195

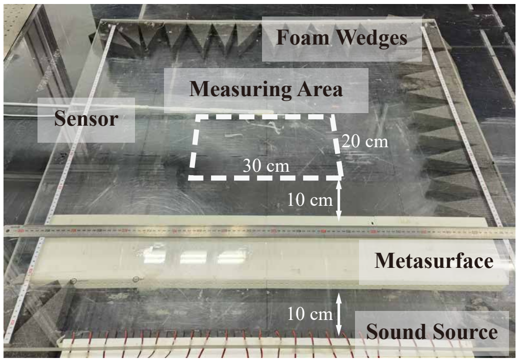

FIG. 5. Experimental setup.

the white dashed box and is depicted in Fig. 6(b). The experimental field in the corresponding measuring area is depicted in Fig. 6(c) and is normalized by the average $|p_0^{\mathrm{Exp}}|$. The exterior-field calculation is defined by an integration along the black dashed line in Fig. 6(b) as

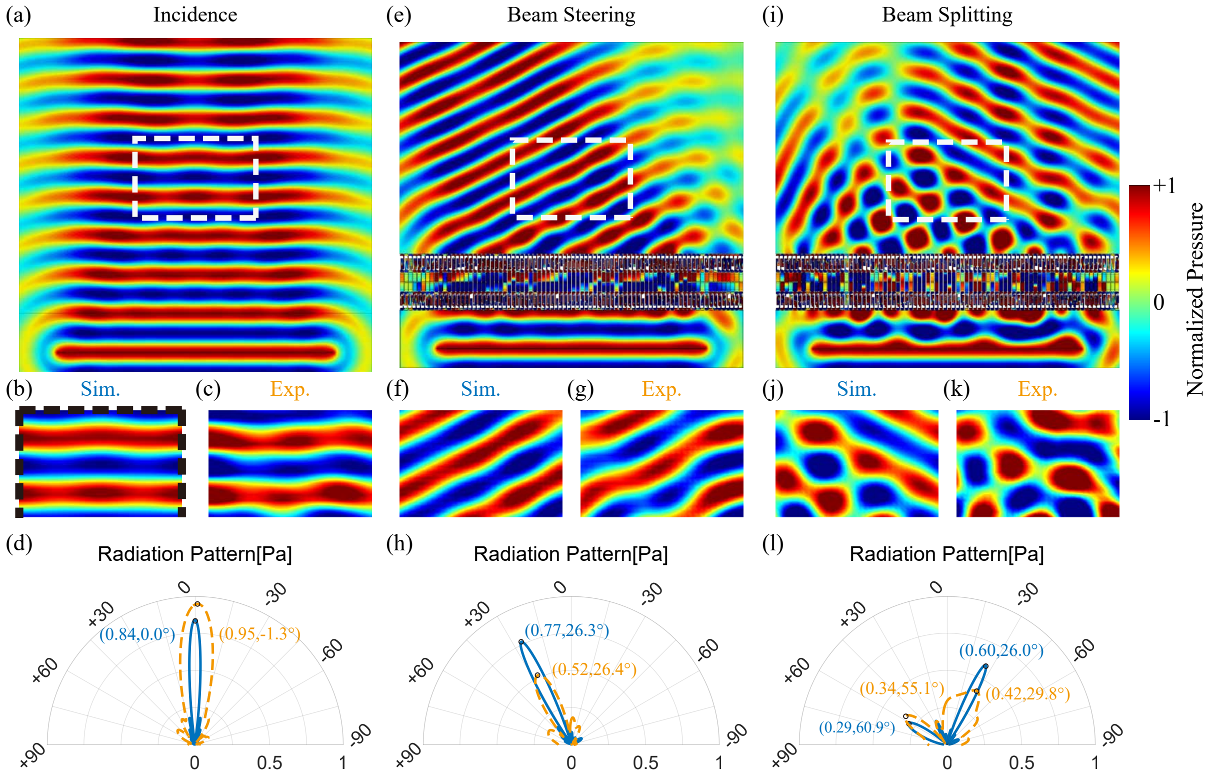

FIG. 6. Comparison of simulation and experimental results. (a) Simulation result for incident plane wave is excited by a finite width beam. The acoustic field of incident plane wave in the measured area is given for (b) numerical simulation and (c) experiment and (d) their radiation pattern. (e) Simulation result for Case 1 is given for the metasurface excited by a finite width beam. The acoustic field of Case 1 in the measured area is given for (f) numerical simulation and (g) experiment and (h) corresponding radiation pattern. (i) Simulation result for Case 3 is given for the metasurface excited by a finite width beam. The acoustic field of Case 3 in the measured area is given for (j) numerical simulation and (k) experiment and (l) corresponding radiation pattern.

$$p_{\mathrm{ext}}\left(\mathbf{R}\right) = -\frac{1-\imath}{4\sqrt{\pi k_0}} \int_{S_{(\mathbf{r})}} e^{\imath k_0 (\mathbf{r}\,\cdot\,\mathbf{R})/|\mathbf{R}|} \left(\nabla p\left(\mathbf{r}\right) - \imath k_0 p\left(\mathbf{r}\right)\frac{\mathbf{R}}{|\mathbf{R}|}\right) \cdot (-\mathbf{n}) . \tag{26}$$

where $\mathbf{r}$ and $\mathbf{R}$ are the vectors from the midpoint of the metasurface to a point on the dashed line and a point in the exterior field, respectively. The amplitudes of the exterior field $|p_{\mathrm{ext}}|$ in simulation and experiment are plotted in Fig. 6 (d) with the blue solid line and the orange dashed line, respectively. It can be observed that the experimental incident field is as flat as in the simulation and is oriented at 0°, which validates the experimental setup. Inserting the beam-steering metasurface, the numerical results are shown in Figs. 6(e,f), respectively. The experimental result is depicted in Fig. 6(g) and is normalized to the same reference values $|p_0^{\mathrm{Exp}}|$ as for Fig. 6(c). The amplitude of the exterior field $|p_{\mathrm{ext}}|$ is plotted in Fig. 6(h) with the same configuration as Fig. 6(d). Therefore the experimental efficiency of the designed metasurfaces can be directly evaluated from a comparison with simulation results. Repeating the above process, a comparison of numerical and experimental results for the beam-splitting metasurface is given in Figs. 6(e-h).

[1] A. Díaz-Rubio and S. A. Tretyakov, Acoustic metasurfaces for scattering-free anomalous reflection and refraction, Physical Review B **96**, 125409 (2017).

[2] A. Díaz-Rubio, J. Li, C. Shen, S. A. Cummer, and S. A. Tretyakov, Power flow–conformal metamirrors for engineering wave reflections, Science Advances **5**, eaau7288 (2019).

[3] Y.-Z. Tian, X.-L. Tang, Y.-F. Wang, V. Laude, and Y.-

S. Wang, Annular acoustic impedance metasurfaces for encrypted information storage, Physical Review Applied **20**, 044053 (2023).
[4] J. Li, A. Song, and S. A. Cummer, Bianisotropic acoustic metasurface for surface-wave-enhanced wavefront transformation, Physical Review Applied **14**, 044012 (2020).
[5] J. Li, C. Shen, A. Díaz-Rubio, S. A. Tretyakov, and S. A. Cummer, Systematic design and experimental demonstration of bianisotropic metasurfaces for scattering-free manipulation of acoustic wavefronts, Nature Communications **9**, 1 (2018).
